# Water Quality in Surface Water: A Preliminary Assessment of Heavy Metal Contamination of the Mashavera River, Georgia

**DOI:** 10.3390/ijerph15040621

**Published:** 2018-03-28

**Authors:** Sisira S. Withanachchi, Giorgi Ghambashidze, Ilia Kunchulia, Teo Urushadze, Angelika Ploeger

**Affiliations:** 1 Department of Organic Food Quality and Food Culture, Faculty of Organic Agricultural Sciences, University of Kassel, 37213 Witzenhausen, Germany; a.ploeger@uni-kassel.de; 2 School of Agricultural and Natural Sciences, Agricultural University of Georgia, Tbilisi 0159, Georgia; g.ghambashidze@agruni.edu.ge (G.G.); t.urushadze@agruni.edu.ge (T.U.); 3 Michail Sabashvili Institute of Soil Science, Agrochemistry and Melioration, Agricultural University of Georgia, Tbilisi 0159, Georgia; ikunc2014@agruni.edu.ge

**Keywords:** wastewater discharge, sediment extraction, mining, food system, water pollution, public health

## Abstract

Water quality contamination by heavy metal pollution has severe effects on public health. In the Mashavera River Basin, an important agricultural area for the national food system in Georgia (e.g., vegetable, dairy and wine production), water contamination has multiple influences on the regional and country-wide health. With new industrial activities in the region, sediment extraction, and discharge of untreated wastewater into the river, its tributaries and irrigation canals, a comprehensive study of water quality was greatly needed. This study examined sediment and water samples from 17 sampling sites in the Mashavera River Basin during the high and low precipitation seasons. The results were characterized utilizing the Geo-accumulation Index (I_geo_), Enrichment Factor (EF), Pollution Load index (PLI), Contamination Factor (CF) and Metal Index (MI). According to the CFs, Cu > Cd > Zn > Pb > Fe > Mn > Ni > Cr > Hg is the descending order for the content of all observed heavy metals in sediments collected in both seasons. Fe and As were additionally examined in water samples. Overall, As, Cd and Pb, all highly toxic elements, were found in high concentrations in downstream sample sites. According to these results, comprehensive monitoring with narrow intervals between sampling dates, more sample sites along all waterways, and proximate observation of multiple trace metal elements are highly recommended. Moreover, as the part of the water quality governance system, an immediate and sustainable collective action by all stakeholders to control the pollution level is highly recommended, as this issue is linked to the security of the national food system and poses a local public health risk.

## 1. Introduction

The United Nations Agenda 2030 and Sustainable Development Goals (SDGs) clearly emphasize the aim to “by 2030, improve water quality by reducing pollution, eliminating dumping and minimizing release of hazardous chemicals and materials, halving the proportion of untreated wastewater and substantially increasing recycling and safe reuse globally” [1]. This is one of the key target of SDG 6: “Ensure availability and sustainable management of water and sanitation for all” (Target 6.3). One of the key objectives of Integrated Water Resource Management (IWRM) and the European Union Water Framework Directive (EU WFD) is to ensure good water quality in all water bodies [2,3]. Heavy metal pollution is considered to be one of the most pronounced issues threatening water quality [4,5,6]. Industrial activities [5], municipal wastewater discharge [7], unsustainable agricultural practices [8], and traffic activities [9] all lead to heavy metal pollution of ecosystems.

There are multiple causes for the water contamination in the Mashavera River Basin, which has been identified as one of the polluted rivers in Georgia [8]. The mining industries in the Kvemo Kartli region have been identified as one of major causes for environmental pollution, particularly the heavy metal contamination of water, airborne particles and soil [10,11,12]. Since 1975, the “Madneuli” mining plant has been operating near Kazreti Village under different management bodies [12]. In 2014, a new mining site was opened in the Dmanisi-Bolnisi region by “RMG Gold” with government permission [13]. The mining areas are open-pit mining sites. Currently, feasibility studies and the pre-excavation process are underway for a new gold and copper mining site—Kvemo Bolnisi Copper Gold Project (‘KB’) [14]. In addition, sediment extraction by road construction companies and local building construction companies are observed along the banks of the Mashavera River. Furthermore, there is direct diversion of untreated wastewater from municipal areas and households due to malfunction of the sewage and drainage systems [15]. Outflows from the farmlands to the Mashavera or Poladauri Rivers or their tributaries can also be observed [8]. 

The Mashavera River Basin contributes a considerable amount of agricultural production to the national food system, including a variety of vegetables, dairy products, meat, wine, beans, wheat and maize [16]. Water is mainly supplied to agricultural fields by irrigation canals originating at the Kazretula, Poladauri and Mashavera Rivers [17] and springs. Most of the irrigation canals are open, and farmers create small waterways from the canals to their land. Some farmers, especially those who are adjacent to the Mashavera River in the downstream areas, are directly pumping water from the river to irrigate their farmland. Some people use the Mashavera River and its tributaries as a source of drinking water [18], and a few wells located near the Mashavera River were observed during field observation. These wells are bored into the alluvial deposit of the Poladauri [17] and Mashavera Rivers. According to Müller [19], the heavy metal contamination of groundwater can occur by infiltration of river water into the banks of the river, thus also affecting the quality of drinking water derived from groundwater. Furthermore, cattle, sheep and goats graze openly in the region and drink water from the Mashavera River and other water canals in the catchment area. Some members of the local communities are even fishing in the Mashavera River and its tributaries for household consumption. The heavy metal contamination of the air, water and soil ultimately impacts the national food system as well as the health of residents of the region [17]. Therefore, extensive study of the concentration and circulation of heavy metals in the river system is important. The key objective of this research paper is to assess the heavy metal concentration in sediments and surface waters of the Mashavera River Basin. Moreover, searching for indications of the negative impacts of poor water quality on the local agriculture is the secondary objective of this study. 

## 2. Materials and Methods

### 2.1. Study Area

This study was conducted as part of a collaborative research project that scrutinizes the multiple impacts of water quality on food security in the Republic of Georgia [20]. The study area included the middle reach to downstream section of the Mashavera River and its tributaries, including the Poladauri and Kazretula Rivers. The length of the Mashavera River is 66 km and the total area of the water basin is 1300 km^2^ [8]. The Mashavera River joins the Khrami River and then the Mtkvari River in Azerbaijan before flowing into the Caspian Sea. The sample locations were situated between latitudes 41°37′–41°44′ N and longitudes 44°38′–44°51′ E. The altitude range was between 410 m and 766 m above the sea level. 

**Figure 1:**
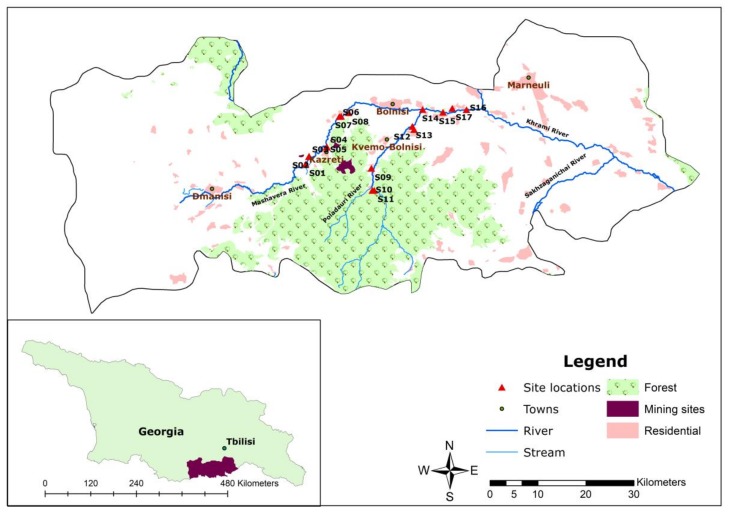
Map of the study area (Authors’ illustration). GIS Data sources: 1. Administrative boundaries [24] and 2. Land-use data and stream data [25].

In terms of political-administrative spatiality, the research area was located in Dmanisi, Bolnisi and Marneuli municipalities in the Kvemo Kartli region of southeastern Georgia (Figure 1). The region is characterized by a semi-arid climate [21]. The average annual precipitation is 500 mm. Compared to other regions in Georgia, it is categorized as a low-lying area. The average temperature is 12 °C, while the coldest month (January) has an average temperature around 0 °C, and the hottest month (July) has an average temperature around 23.9 °C. However, an increase in the average air temperature has been observed [22]. The upstream areas of the Mashavera river catchment and Poladauri River, one of the Mashavera’s main tributaries, have relatively high precipitation and humid climatic conditions [23]. 

### 2.2. Sample Collection 

The preliminary research study was conducted in September 2015 to identify the sampling locations. A description of the sampling sites is given in Table 1. The possible wastewater outflows, tributaries, irrigation canals, upstream locations and other human-ecological interactions were taken into account in choosing the targeted sampling locations [26]. At each of the 17 identified sample sites, the pH and electric conductivity (EC) were measured during the high precipitation season (HPS) in May 2016 and the low precipitation season (LPS) in September 2016 using a YSI ProDSS pH meter (YSI Inc./Xylem, Rye Brook, NY, USA) and ATC, Etekcity EC meter (HM Digital TDS-EZ, Suite A Anaheim, CA, USA). Additionally, water samples were collected from all 17 locations in both seasons according to the methods of Singare et al. [27]: 3 L of unfiltered water samples were collected at three times during the day (i.e., morning, midday and evening) in bottles rinsed by distilled water. The dissolved concentrations of heavy metals have been shown to vary considerably throughout the day due to anthropogenic and ecological factors [28,29]. Thus, water sample collection three times a day is recommended to observe diurnal (24 h) concentration patterns of heavy metals [29,30,31]. Nitric acid (pH ∼ 2, 68%) was then added to the samples and they were transferred to the laboratory in Tbilisi, Georgia while kept below 4 °C in a portable cooler [5,6,27]. The total number of water samples was 102 over both seasons. By following the guidelines from the Handbook for Sediment Quality Assessment by Simpson et al. from the Commonwealth Scientific and Industrial Research Organisation (CSIRO) in Australia [26], 17 samples were collected in each season of the top 5 cm of sediment on the banks of the waterways at locations corresponding to the water sampling sites. Suitable access points were found and sediment samples were gathered using a plastic garden-hand shovel and then stored in labeled glass bottles rinsed with distilled water. All glass bottles were kept below 4 °C in a portable cooler during transport to the laboratory in Tbilisi, Georgia. 

### 2.3. Sample Preparation and Instrumental Analysis

Each water sample was filtered through filter paper (2 μm), poured into labeled beakers and placed in an 80 °C drying oven (Heraeus Thermo Scientific, Waltham, MA USA) for deliberate evaporation until a volume of 50 mL was achieved. The time required for evaporation of each sample depended on the amount of dissolved solutions. Four mL of sulfuric acid was added to each 50 mL sample [5]. Then, each sample was digested for 3 min and 10 mL of hydrogen peroxide (H_2_O_2_) was added. The samples were then heated until completion of oxidation. Each final sample was then poured into a glass tube for cooling [5]. All finalized samples were analyzed using AAS (Atomic Absorption Spectrometry—Analyst 700, Perkin Elmer, Rodgau, Germany) in the Heinz Fehr Laboratory of Ecological Agriculture and Nature Conservation at the Agricultural University of Georgia. 

Sediment samples were dried in the sand oven (Thermo Fisher, Stadt Hennigsdorf, Germany) under controlled and constant temperature (80 °C) [32]. Dried samples were then sieved using a 53 μm sieve (Fritsch, Idar-Oberstein, Germany). Thirty g from each sieved sediment sample was packed into an airtight storage bag and then transported to Germany. EC and pH of each sample was tested in the soil analysis laboratory at the University of Kassel, Germany. For pH testing, a solution was prepared for each sediment sample based on 1:2.5 ratio (10 g sediment and 25 mL Milli-Q Water (Millipore Corporation, Burlington, MA, USA)) [6] and then the pH was measured (SCOTT PS/ISEPro Lab 1000, Xylem, Rye Brook, NY, USA). For testing EC, a solution was prepared based on 1:6 ratio (5 g sediment and 30 mL Milli-Q Water (Millipore Corporation, Burlington, MA, USA)) [6] and then EC was measured (QCOND 2200, VWR, Hannover, Germany)). At the Hess State Laboratory in Kassel, Germany, 3 g from each sample were treated with aqua regia (HNO_3_ + 3HCl) in a closed Teflon vessel (120 mL) and then samples were digested in the microwave digestion system. After filtration through a 0.45 μm syringe filter, 50 mL of each sample was stored in a tube. Each sample was analyzed by ICP-MS (Inductively Coupled Plasma Mass Spectrometry-NexION 300D, PerkinElmer Inc., Waltham, MA, USA) and ICP OES (Inductively Coupled Plasma Optical Emission Spectrometry—Optima™ 8300, PerkinElmer Inc., Waltham, MA, USA). Hg was analyzed by CV-AAS (Cold vapor atomic absorption spectroscopy). All analyses were carried out according to the international standards, namely DIN EN ISO 17294, 12338 and 11885. 

### 2.4. Analytical Methods 

Quality indices, as an amalgamated measure of several individual quality characteristics, assist by providing an overview of the environmental quality status [4]. Duodu et al. [33] emphasize that the application of multiple indices to assess heavy metals in a research location overcomes the limitations of applying only a single index. Data from sediment samples were analyzed according to the geo-accumulation index (I_geo_), enrichment factor (EF), pollution load index (PLI) and contamination factor (CF). In the sediment analysis, Pb, Cd, Cu, Cr, Ni, Hg, Zn, Mn and Fe content were tested. Global average shale values were used to determine typical background heavy metal concentrations to assess geochemical data [6,32]. This is a vital comparison to make in any assessment of the anthropogenic heavy metal pollution in sediments of river ecosystems [34]. The global average shale values for Pb, Cd, Cu, Cr, Ni, Hg, Zn, Mn and Fe were taken from the research paper of Turekian and Wedepohl [35]. The metal index (MI) was applied to analyze the metal content of the water samples in comparison to the maximum allowable concentrations from the Georgian National Water Regulation. Pb, Cd, Cu, Cr, Ni, Zn, Hg, Mn and As were analyzed in water samples.

#### 2.4.1. Geo-Accumulation Index

The Geo-accumulation Index (I_geo_), which was first formulated by Müller [19], is a widely applied index to calculate and assess the pollution in soil, dust or sediments [36,37]. In the I_geo_, the existing status of the heavy metals is evaluated in comparison with pre-industrial levels [19] according to the following calculation:

I_geo_ = log_2_ [C_n_/1.5 B_n_] (1)

where C_n_ is the measured concentration of each heavy metal in the sediments and B_n_ is the background value for each corresponding heavy metal. The constant 1.5 is applied as the background matrix correction factor [37]. Based on Müller’s [19] explanation, I_geo_ has six classes ranging from 0 (non-contaminated) to 6 (extremely polluted) (Table 2). 

#### 2.4.2. Enrichment Factor (EF)

The Enrichment Factor (EF) is another widely applied index to determine the contamination in different environments [32] and can be used to postulate the trend of geochemical characteristics athwart a geographic area [38,39]. Various elements can be used in the calculation to represent normal background values, such as Al [6], Fe [40,41], Mn [42], Li [43], Sc or Zr [44]. Fe has a high natural concentration compared to other heavy metals (dominant input) and less possibility to be enriched by anthropogenic causes [39,41,45] This research used Fe for the reference background values as it is an immobile element in the natural resources of the study area.

The formula for EF based on Buat-Menard and Chesselet [46] is:

(2) $$\mathrm{EF}=\frac{\left(\frac{M}{Fe}\right)sample}{\;\left(\frac{M}{Fe}\right)background}$$

where (*M*/*Fe*)*_sample_* is the ratio of the concentration of the examined heavy metal to Fe in the observed the sample, and (*M*/*Fe*)*_background_* is the ratio of the concentration of the examined heavy metal to Fe at normal background values. The scale of the enrichment factor (EF) is given in Table 2. 

#### 2.4.3. Contamination Factor (CF) and Pollution Load Index (PLI) 

The Contamination Factor (CF) is calculated as the ratio of the concentration of a heavy metal in the observed sample (*C_i_*) and the background level of the same heavy metal (*C_b_*) [47]. Though CF is relatively simply to calculate, one constraint is that CF does not consider lithogenic and sedimentary inputs of the observed heavy metal [33,48]. The corresponding equation is [33]:

(3) $$\mathrm{CF}=\frac{C_{i}}{C_{b}}$$

where *C_i_* is the measured concentration of each heavy metal in the sediments and *C_b_* is the background level for each corresponding heavy metal. 

The Pollution Load Index (PLI) demonstrates the general contamination level by considering the overall toxicity of all observed heavy metals [6]. According to Tomlinson et al. [49], the PLI is the nth root of the aggregation of the contamination factors in the research area (Equation (4)). PLI provides a temporal and spatial overall indication of pollution in the environment, which can be of assistance in environmental governance [41,50]. The PLI is calculated according to the following formula:

(4) $$\mathrm{PLI}=\sqrt[{n}]{CF_{1}}\times \;CF_{2\;}\times \;CF_{3}\cdots CF_{n}$$

where CF is the Contamination Factor and *n* is the total number of observed heavy metals. The scale of PLI is noted in Table 2. 

#### 2.4.4. Metal Index

The Metal Index (*MI*) was applied to analyze the quality of drinking water [52], canal water [53] and river water [54,55]. Based on Tamasi and Cini [56], *MI* is calculated as follows:

(5) $$MI=\overset{N}{\underset{i=1}{\sum }}\;\frac{C_{i}}{{\left(\mathrm{MAC}\right)}_{i}}$$

where *C_i_* is the concentration of each heavy metal in each sample, and MAC is the maximum allowable concentration based on the standards of the country. *MI* is considered the cotemporary aggregate tendency of the quality status [53], which provides an overall understanding of the water quality for policymakers as well as the community. *MI* > 1 is the warning threshold [54], even though the *C_i_* may be less than (MAC)*_i_* for certain metals [56]. Table 3 shows the water quality classification categories of *MI*. 

### 2.5. Statistical and Graphical Analysis

The data were statistically analyzed using SPSS version 24.0 (IBM, Armonk, NY, USA), Excel (2013) (Microsoft, Redmond, WA, USA) and Origin 8.5.1 (OriginLab Corporation Northampton, MA, USA). ArcMap 10.1 (ESRI, Redlands, CA, USA) was used for mapping the study area. 

## 3. Results and Discussion 

### 3.1. Chemical Properties 

EC and pH, the chemical parameters measured in the laboratory as well as at the sample sites (in situ), are given in Table 4. Except sample site 9, all other sites were in the pH range of 7 to 9 (neutral to alkaline conditions). Sample site 9 had acidic water and sediment samples in both seasons. According to Fondriest Fundamentals of environmental measurements [57], the water samples at site 9 can be categorized as industrial wastewater based on their EC values (EC = 29,952 µS/cm and 11,115 µS/cm in the wet and dry season, respectively).

### 3.2. Heavy Metal Concentrations in Sediments 

In this research study, the sediments were analyzed for Pb, Cd, Cu, Ni, Hg, Zn, Mn and Fe. In the Table 5, the heavy metal data for the 17 sample sites for the HPS (May) and LPS (September) are listed. Observing the mean concentrations of heavy metals in the sediments, Fe > Mn > Zn > Cu > Pb > Cr > Ni > Cd > Hg is the descending order of element concentrations. Cd had high concentrations in the downstream samples sites (S 12, 13, 14, 15 and 16) compared to the upstream sites, except Site 4. Pb had relatively high concentrations at the Mashavera River sites, namely Site 3 (186 mg/kg in the HPS and 130 mg/kg in the LPS), Site 4 (94.9 mg/kg in the HPS and 73 mg/kg in the LPS) and Site 17 (63.4 mg/kg in the HPS and 35.3 mg/kg in the LPS). Sample site 12 had high concentrations of Cd in both seasons (8.35 mg/kg and 6.11 mg/kg, respectively) compared to other sites. Hg had low concentrations at all samples sites. Some samples sites showed concentrations higher than the recommended levels for Pb, Cd, Cu, Ni and Zn according to the Sediment Toxicity Reference Values (STRVs). However, the index analysis is needed to observe these trends in a more analytical way. 

### 3.3. Index Analysis of Heavy Metal Pollution in Sediments 

**Figure 2:**
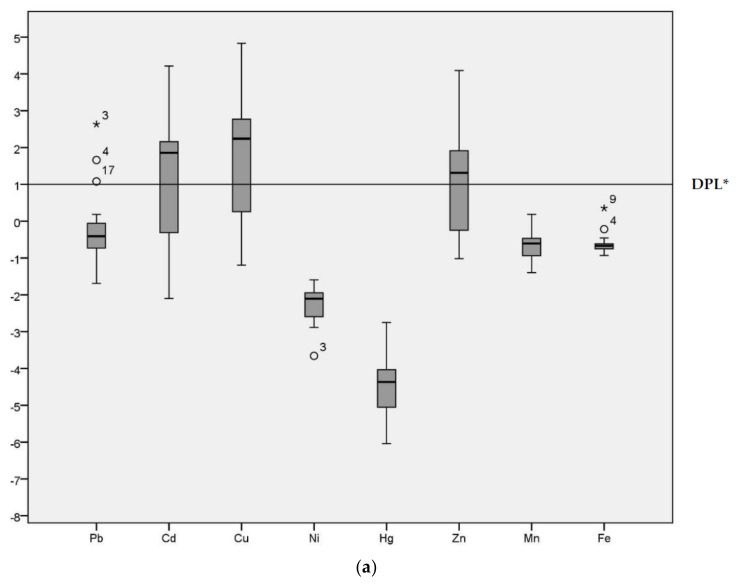

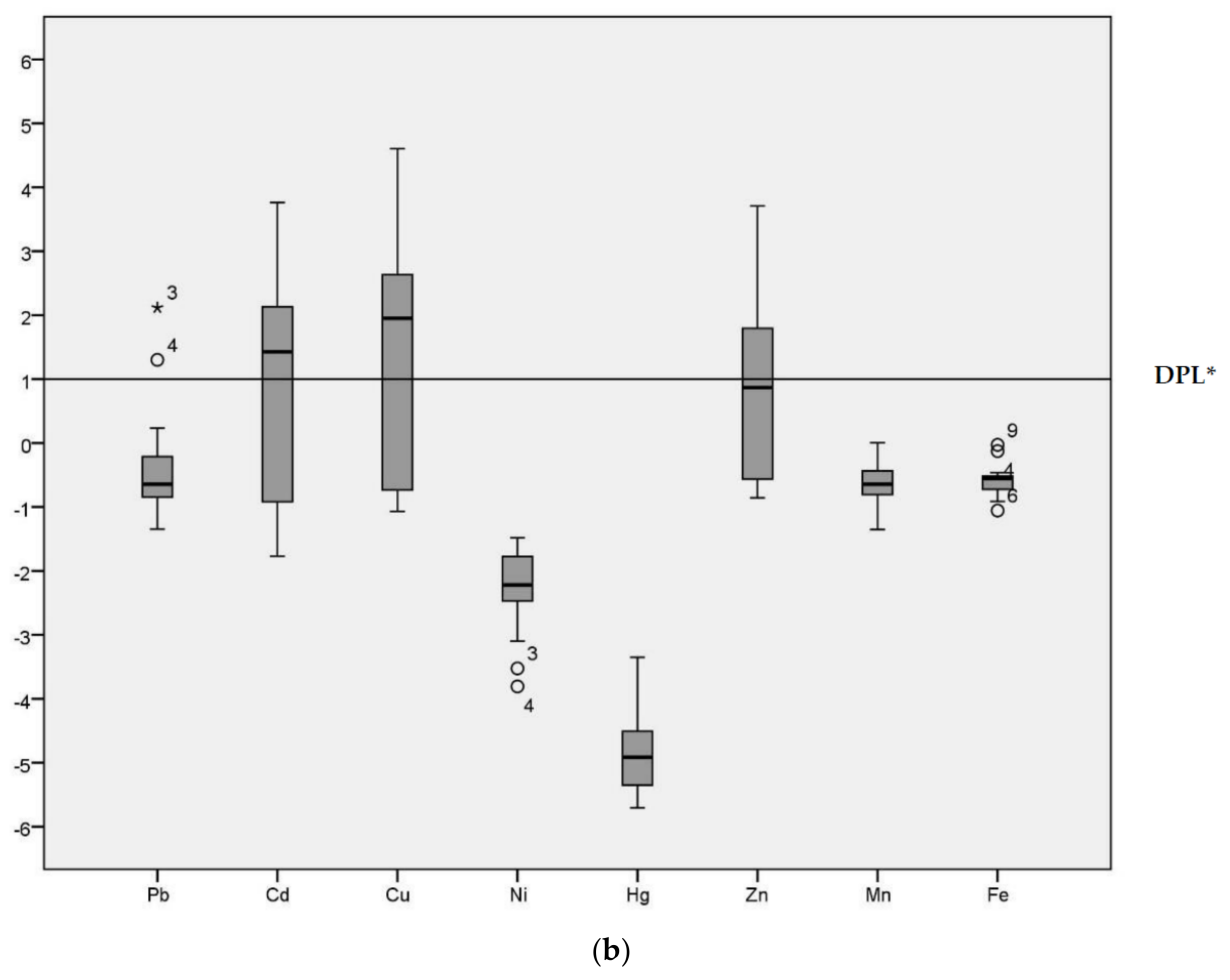
Geo-accumulation Index (I_geo_) values of heavy metals in sediments of samples sites. (**a**) Geo-accumulation (I_geo_) in HPS; (**b**) I_geo_ in LPS; * DPL: demarcation of pollution level.

Geo-accumulation (I_geo_) values are given in the Figure 2 for the HPS and LPS separately. Although the majority of I_geo_ values for Pb were below Class 1 in both seasons, sample site 3 was an extreme outlier (I_geo_ = 2.63 in the HPS and I_geo_ = 2.12 in the LPS) in both seasons. Sample site 4 (I_geo_ = 1.66 in the HPS and I_geo_ = 1.30 in the LPS) and 17 were also outliers for Pb (I_geo_ = 1.08 in the HPS).

The median for Cd was above the demarcation of pollution level for both seasons. The range of I_geo_ for Cd was −2.10 to 4.21 in the HPS and −1.77 to 3.76 in the LPS. In particular, sample site 12 (irrigation canal) was categorized as I_geo_ Class 5 (I_geo_ = 4.21) in the HPS and I_geo_ Class 4 (I_geo_ = 3.76) in the LPS. Cu had an I_geo_ range from −1.19 to 4.38 and −1.07 to 4.60 during HPS and LPS, respectively, indicating conditions ranging from non-polluted to strongly/extremely polluted. The median for Cu was above the demarcation of pollution level for both seasons. Ni, Hg, Mn and Fe all had predominantly negative I_geo_ values, with median values below the demarcation of pollution level for both seasons. Zn was above threshold values in the HPS. The median for Cu was above the demarcation of pollution level only in the HPS, with I_geo_ ranging from −1.02 to 4.09. In the LPS, conditions fluctuated between non-polluted (−0.86) to highly polluted (3.71). I_geo_ analysis enables identification of the degree of contamination from the heavy metals [6] and the variation in the pollution level across different sample locations [32] in the study area.

**Figure 3:**
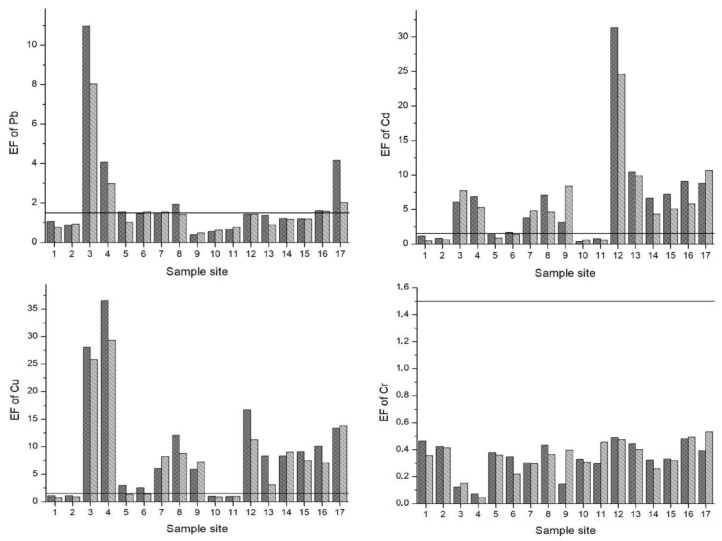

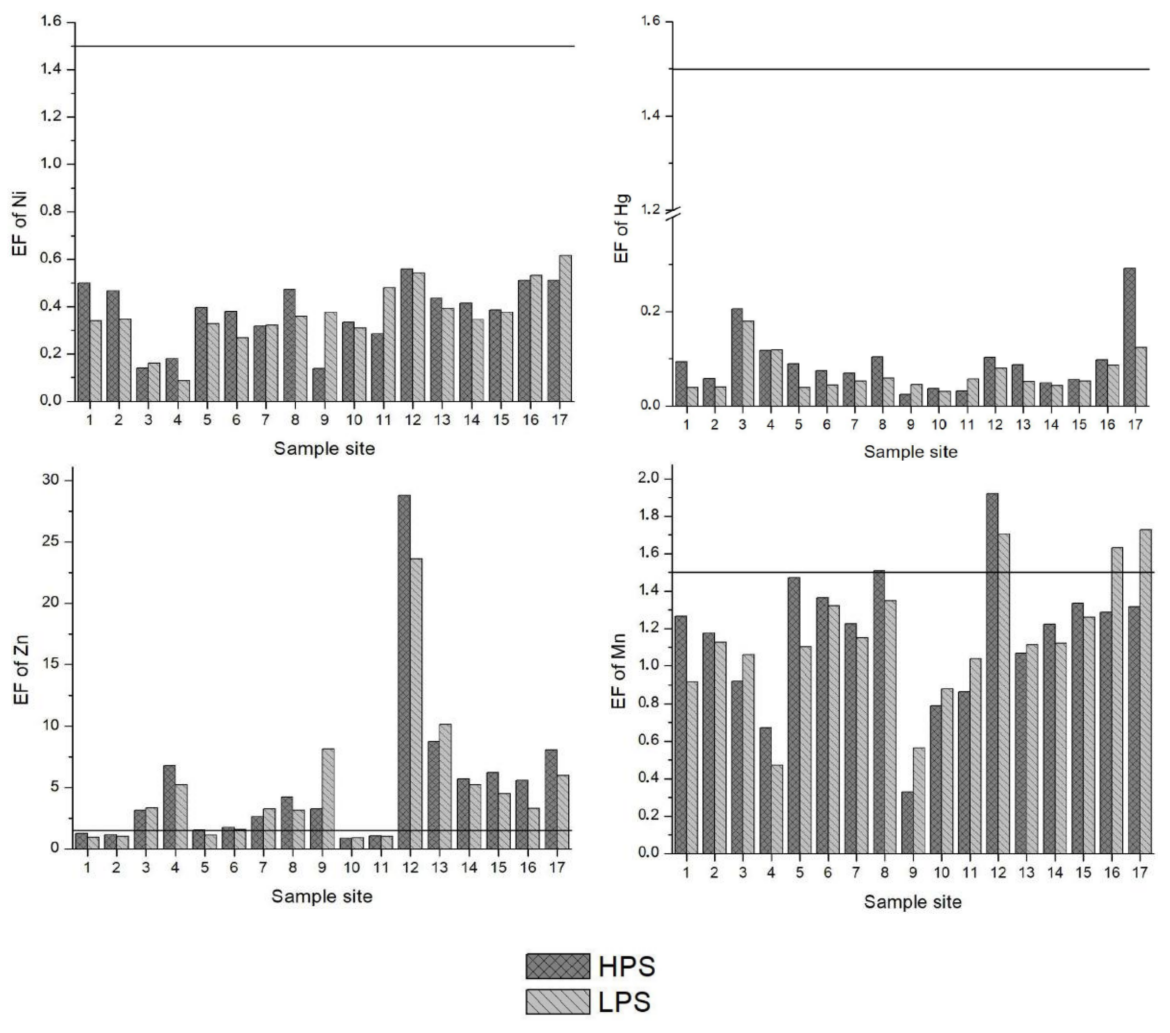
Enrichment Factors (EF) of heavy metals in sediments of samples sites. A value of 1.5 (line) was set as the threshold determining if enrichment levels signified anthropogenic influence.

The Enrichment Factor (EF) is given in Figure 3. Based on the study of Zhang and Liu [56], an EF of 1.5 is designated as the threshold value to separate natural levels of crustal enrichment in heavy metals (1.5 < EF) from levels caused by anthropogenic intervention (1.5 > EF). Pb, Cd, Cu and Zn were above the 1.5 threshold value at many of the sample sites. Specifically, EFs for Pb were in the range of moderate to significant enrichment at sample sites 3, 4 and 17. Except sample sites 1, 2, 10 and 11 for both seasons and 5 and 6 for the LPS, the EFs for Cd oscillated between moderate to very high enrichment at all other sites. In particular, sample site 12 (the irrigation canal in Vanati village) had very high enrichment for both seasons (EF = 31 and 24 in the HPS and LPS, respectively). Also, the EFs for Zn ranged from normal levels of mineral enrichment to very high enrichment. The downstream areas of Mashavera River and Poladauri River showed considerable anthropogenic enrichment for Cd, Cu and Zn and some Pb enrichment in downstream irrigation canals.

**Figure 4:**
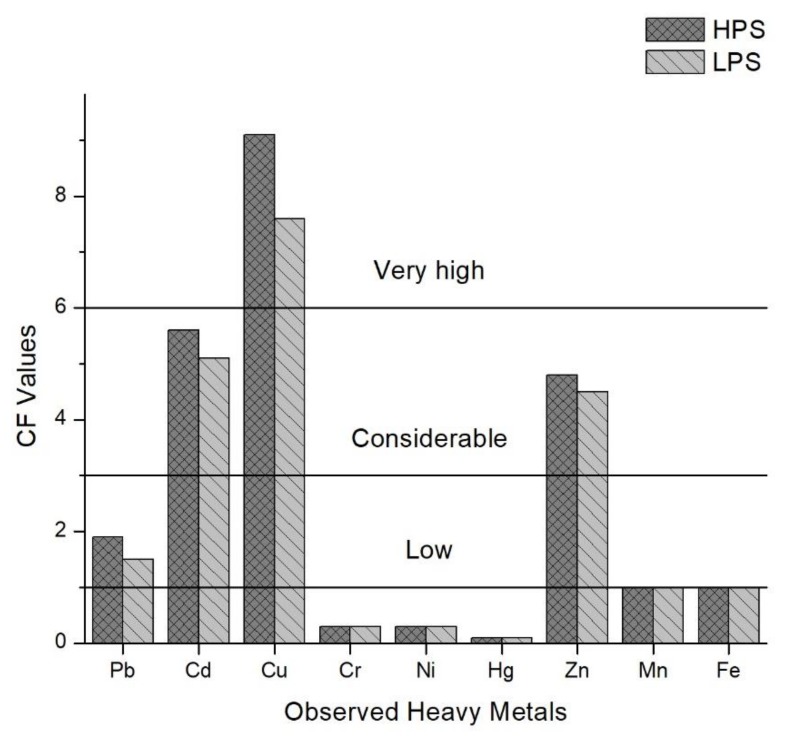
Contamination Factors (CF) of heavy metals in sediments of the samples sites.

Based on the study of Islam et al. [6], the mean values of the Contamination Factors (CF) for all observed heavy metals in the sediment samples were calculated in this study (Figure 4). The mean values for the HPS were 1.9 (Pb), 5.6 (Cd), 9.1 (Cu), 0.3 (Cr), 0.3 (Ni), 0.1 (Hg), 4.8 (Zn), 1.0 (Mn) and 1.0 (Fe). 

**Figure 5:**
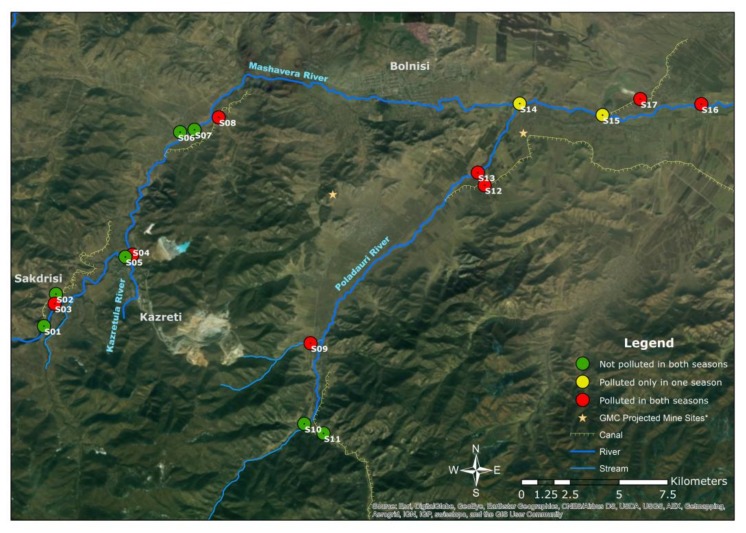
Spatial assessment of Pollution Load Index (PLI) for both seasons (Authors’ illustration). * GMC projected mine sites [59]; GIS Data source: 1. Land-use data and stream data [21] and 2. Base map from ESRI satellite image layer (ArcMap).

In the LPS, mean values were 1.5 (Pb), 5.1 (Cd), 7.6 (Cu), 0.3 (Cr), 0.3 (Ni), 0.1 (Hg), 4.5 (Zn), 1.0 (Mn) and 1.0 (Fe). Cu > Cd > Zn > Pb > Fe > Mn > Ni > Cr > Hg is the descending order of contamination factors (CF) for all observed heavy metals in both season. The Pollution Load Index (PLI), given for both seasons in Figure 5, delivers the overall outlook of the contamination spatially and temporally [41]. Sample sites 3, 4, 9, 12, 13, 16 and 17 were contaminated for both seasons, and sample sites 14 and 15 were polluted in the HPS.

### 3.4. Correlation among Individual Heavy Metals and PLI in the Sediment Samples

The calculation of the correlation matrix between individual heavy metals demonstrates the relationships between each of the elements that could be detected. The basis for these correlations could be geochemical relationships or the common sources [60,61,62,63] as well as mutual dependences or identical behavior in the transportation process [62]. Spearman correlation analysis [63] was applied to analyze the data. Table 6 illustrates the correlation matrix for the HPS, whereas Table 7 gives the LPS matrix. In HPS, Pb had strong and significant (*p* < 0.01) correlations with Cd (*rs* = 0.620), Cu (*rs* = 0.733), Hg (*rs* = 0.903), and Zn (*rs* = 0.529). Cd was additionally significantly correlated with Cu (*rs* = 0.765, *p* < 0.01), Zn (*rs* = 0.939, *p* < 0.01) and Hg (*rs* = 0.569, *p* < 0.05). Cu also showed a significant correlation (*p* < 0.01) with Hg (*rs* = 0.652) and Zn (*rs* = 0.806), and Cr had a positive significant correlation (*p* < 0.01) with Ni (*rs* = 0.951) and Mn (*rs* = 0.735). Ni was additionally strongly positively correlated (*p* < 0.01) with Mn (*rs* = 0.792). Hg was significantly correlated (*p* < 0.05) with Zn (*rs* = 0.495) as well. In the LPS, Pb significantly correlated (*p* < 0.01) with Cu (*rs* = 0.679) and Hg (*rs* = 0.645). Cd strongly and significantly positively correlated (*p* < 0.01) with Cu (*rs* = 0.826), Hg (*rs* = 0.732) and Zn (*rs* = 0.909). Cu additionally had a significant correlation (*p* < 0.01) with Hg (*rs* = 0.792) and Zn (*rs* = 0.750). There was a significant correlation of Cr also with Ni (*rs* = 0.9689, *p* < 0.01) and Mn (*rs* = 0.561, *p* < 0.05). Ni had a positive significant correlation (*p* < 0.01) only with Mn (*rs* = 0.613), and Hg significantly correlated (*p* < 0.05) with Zn (*rs* = 0.537). Fe did not show a correlation with any other heavy metals. There were seasonal differences between the correlation of Pb and Cd as well as between Pb and Zn. Some studies have identified a natural geochemical relationship between Pb and Zn [63,64]. The correlation matrices show that there are strong positive correlations between Pb and Cu, Pb and Hg, Cd and Cu, Cd and Hg, Cu and Hg, Cr and Ni, Cr and Mn, Ni and Mn, and Hg and Zn in both seasons. These correlations among individual heavy metals indicate common sources, mutual dependences and similar behavior in the transportation processes in the research area. These common geochemical behaviors could derive from weathering processes, source-rock surfaces and adsorption phenomena [60]. 

The strong positive correlations between PLI and several heavy metals (Table 6 and Table 7) indicate which metals contribute most strongly to overall pollution [62]. Apart from Cr, Ni and Mn, there are a strong positive correlations between PLI and Pb (*rs* = 0.789, *p* < 0.01 in HPS and *rs* = 0.514, *p* < 0.05 in LPS), Cd (*rs* = 0.926, *p* < 0.01 in HPS and *rs* = 0.939, *p* < 0.01 in LPS), Cu (*rs* = 0.895, *p* < 0.01 in HPS and *rs* = 0.868, *p* < 0.01 in LPS), Hg (*rs* = 0.754, *p* < 0.01 in HPS and *rs* = 0.843, *p* < 0.01 in LPS) and Zn (*rs* = 0.885, *p* < 0.01 in HPS and *rs* = 0.838, *p* < 0.01 in LPS). As discussed above, the PLI indicates the anthropogenic pollution in the research area.

### 3.5. Heavy Metal Concentrations in the Water

As described above, three separate water samples were collected for monitoring from each site in the morning, midday and evening. In Table 8, the mean value of each heavy metal for each sample site is listed. Analyzing the average concentrations at the sample sites, Zn > Cu > As > Ni > Pb > Cr > Cd is the descending order of the concentrations for both seasons. However, there were considerable differences in HPS and LPS for some heavy metals at certain sites. At site 9, the mean concentration of Ni in the LPS (180.3 µg/L), Cu in the HPS (2603.7 µg/L) and LPS (13,157.6 µg/L), Zn in the LPS and HPS (21,912 µg/L and 12,505 µg/L, respectively), and Cd in the HPS (62.5 µg/L) were above all thresholds adopted by Georgian regulation 2001 [65], EU standards [66] and WHO standards [67] The EU standards are based on the European Council Directive 98/83/EC on the quality of water intended for human consumption [67]. These standards are relevant given the multiple uses of Mashavera River for crop and livestock production. The average concentration of As was above all standards at sites 1, 2, 3, 4, 5, 7 and 8 in the LPS. Cd was above the standards in the HPS for site 9 (62.5 µg/L) and 10 (10.1 µg/L). Pb showed high average concentration at site 1 in the HPS (15.8 µg/L) and LPS (11.1 µg/L) as well as levels above the standards at sites 3 and 4 (69.6 µg/L and 62.8 µg/L, respectively, in the LPS).

**Figure 6:**
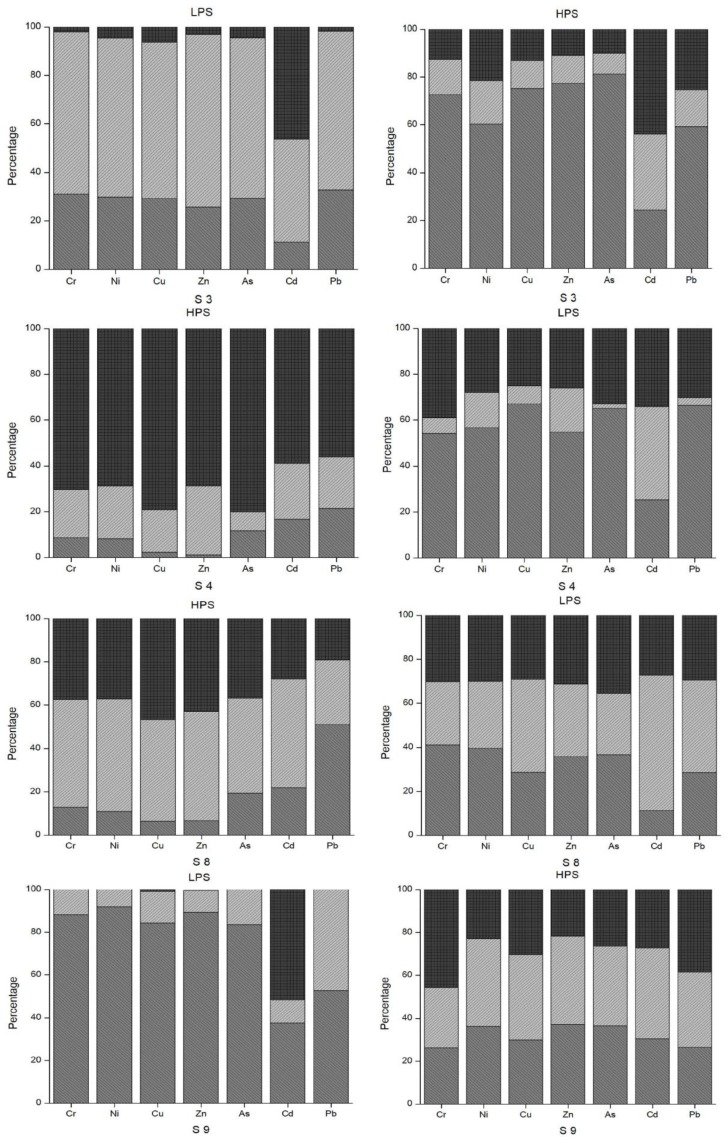

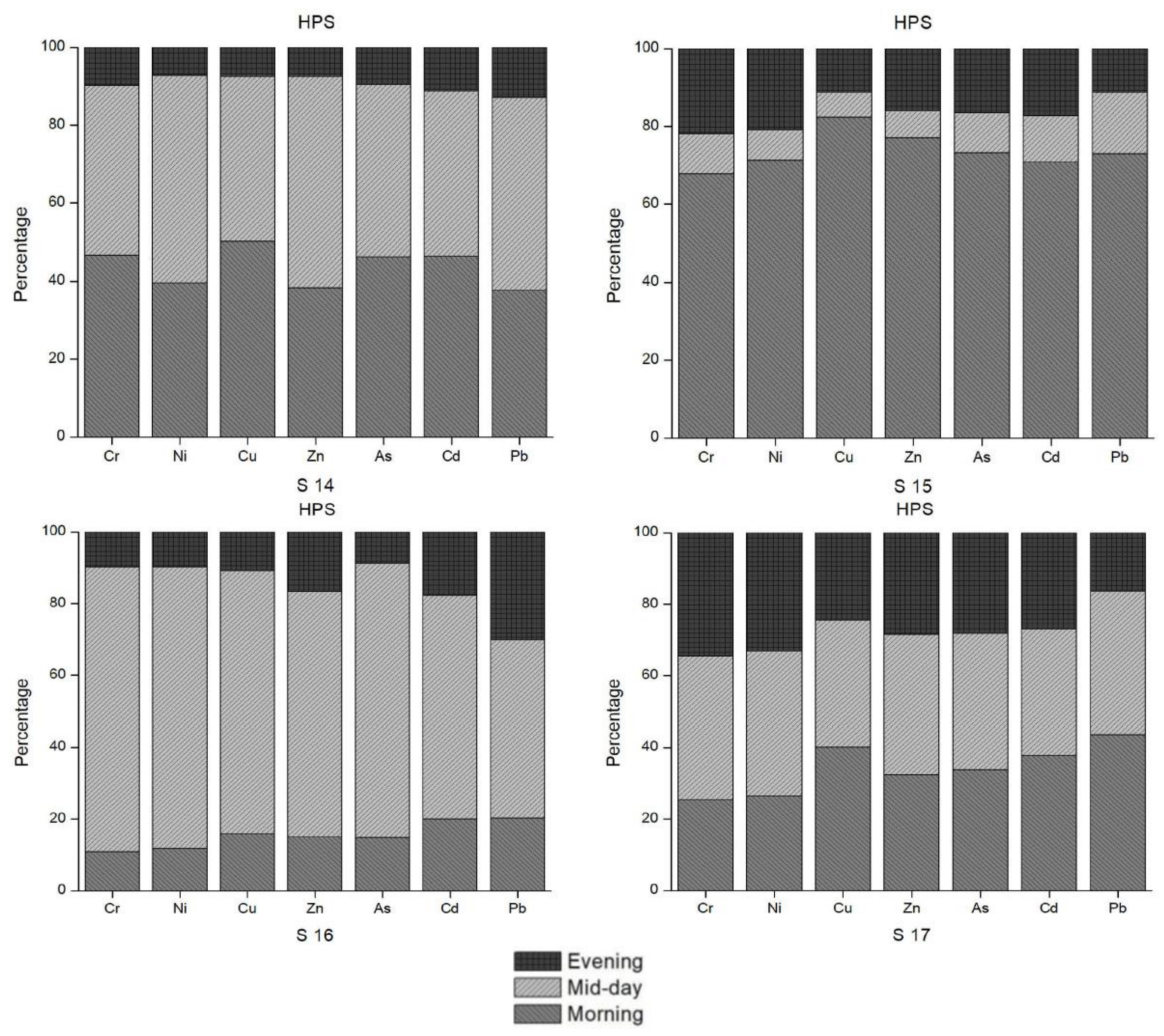
Diurnal trends in heavy metal concentration for selected sample sites.

By observing the raw data (before calculation of mean values), notable oscillations in the concentrations of some heavy metals between morning, day and evening were identified. Figure 6 summarizes these diurnal as well as seasonal changes in the selected sample sites where considerable changes were observed. Based on the field interview data conducted for this study, these changes throughout the day could also be a result of the timing of anthropogenic activities, such as releasing wastewater to the river and canal. Otherwise, physical and chemical reactions in the water bodies could be possible explanations for such diurnal variations [31]. According to Bourg and Bertin [28] as well as Nimick [31], there is a diurnal variation in water temperature, pH, dissolved oxygen content, and concentrations of Zn, Cd and Mn due to photosynthesis. For a solid understanding of diurnal variations in the concentrations of heavy metals, more long-term research has been recommended [28,29]. Our results also support the need for further research to examine the exact causes of these diurnal and seasonal changes.

The Aquatic Toxicity Reference Values (TRVs) indicate the toxicological benchmark for the aquatic habitat, which is applied as an ecological risk assessment (ERA) [59,68]. Concerning the Surface Water Toxicity Reference Values (SWTRVs), Pb values were above the recommended levels in the HPS at all sites except 10, 11, 12, 13 and 16. The concentrations at site 3, 4 and 9 for Cu and Zn and site 3 and 4 for As were also above the TRVs for surface waters. As, Pb and Cd, all highly toxic elements [69], ultimately bioaccumulate in aquatic organisms [70,71]. This can then affect humans through the consumption of fish living in these water bodies [70,71]. During the field observation, a couple of fishermen were encountered at the Mashavera River and open interviews were conducted with them about their fishing practices. They explained that households and local restaurants in the region serve locally-caught river fish as a traditional dish.

### 3.6. Index Analysis of Heavy Metal Pollution in Water

**Figure 7:**
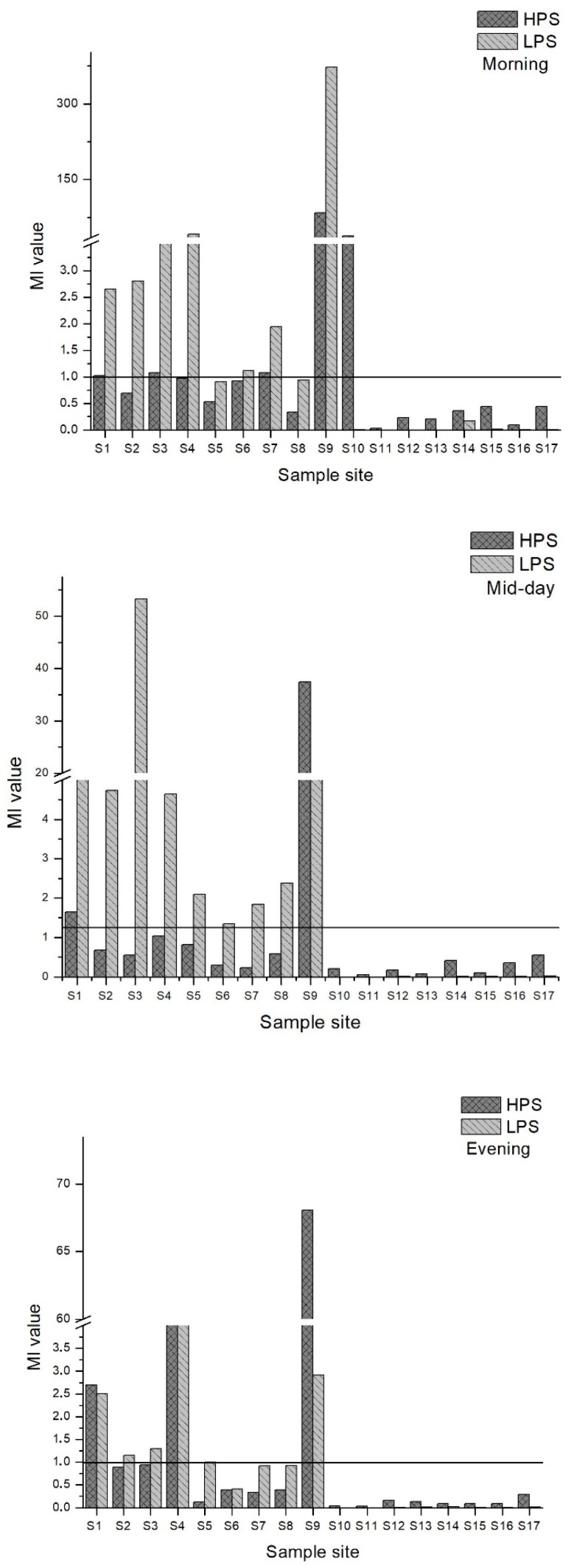
MI values for morning, midday and evening water samples for both seasons.

Heavy metal concentrations of water samples were analyzed using the Metal Index (MI). Figure 7 demonstrates the MI for the morning, midday and evening for both seasons. MI = 1 was set as the threshold value above which there should be a quality warning based on the literature [54]. Site 9 values in HPS and LPS were classified as seriously affected. In the morning samples, MI values were 29 in HPS and 134 in LPS. MI values were 37 in HPS and16 in LPS for the midday samples. In the evening, MI values were 23 for HPS and 1.03 for LPS. Site 4 had a high MI (MI = 41) in the evening samples of LPS. Thus, sample site 4 was in the moderately affected to the seriously affected category for the MI. Site 12 to 17 had low MI values and site 10 and 11, the upstream sites in Poladauri River, were classified as very pure (MI < 0.3). There were low MI values in the downstream sites. 

### 3.7. Previous Studies and the Overall Current Status

There were previously a few research studies that examined the heavy metal pollution of the soil and effect on the food crops in the Mashavera River Basin [8,10,11,12,72,73,74]. Those studies already identified the heavy metal pollution in the area in different mediums. Moreover, the Department of Environmental Pollution Monitoring of the National Environmental Agency (NEA) is monitoring water quality at two sample sites along the Mashavera River [75]. This data is publicly available on the National Environmental Agency website as well as in the annual report. In Table 9, the sediment and water heavy metal concentrations from some of the previous studies and NEA monitoring reports are listed. As Avkopashvili et al. [12] also found, the Mashavera River has a high concentration of Zn and Cd. The focus of the previous studies was on the wastewater outflow into the Kazretula River (sample site 4) [8,10,11,12,72,74].

This research study examined sediments and water samples for the HPS and LPS at 17 sample sites to identify various field characteristics (Table 1). Collectively, all the analysis of this study give a comprehensive overview of the water quality status of the Mashavera River and its tributaries as well as the major irrigation canals in the basin. Sample site 1 at the Mashavera River and the sample sites 10 and 11 were chosen as the upstream control points. These sites indicate relatively less pollution compared to other sites or even an unpolluted status. The overall results from the sediment and water samples for both seasons indicate that there is still considerable outflow of heavy metals through the Kazretula River to the Mashavera River at site 4. 

Figure 6 illustrates the diurnal and seasonal changes in the heavy metal concentrations in the water samples. Site 9 is another outflow of the Madneuli mine sites, ultimately joining the Poladauri River, which shows an alarming level of heavy metal contamination for water and sediment samples. There may be a connection between this water outflow from the mine site and pollution at sample site 12, which was located on the irrigation canal in Vanati village and indicated very high enrichment of Cd and Zn, and significant enrichment of Cu. In interviews, locals noted that there is a substantial color change of the outflow in the HPS and the LPS, which was verified by the field observations. The PLI index clearly shows the extreme level of contamination of these sites in both seasons. Proper monitoring by the responsible governance agency or the mining industry and treatment of water before it enters this tributary are recommended for the betterment of the current condition.

Compared to water samples at the sites, sediment samples clearly demonstrate the high concentrations of heavy metals. The direct enrichment from weathering sources, clay particles in the soil, long-term sedimentation processes from water flow, and dust particles from the air [37,76,77] are possible causes of the high concentrations of toxic elements. Site 3 showed high-level contamination of water as well as sediment. The sediment extraction from the Sakdrisi open-pit mining site could mix with the Mashavera River. The research done by Tamar Manjavidze [78] demonstrates that milk, meat and vegetables make up a high share of the daily diet of the population in Georgia. However, there are considerable regional and seasonal differences in food consumption patterns. The field observations and interviews confirm that this water is being used by farmers for cultivation. The riverine areas are used for grazing livestock, and the field observations revealed that animals are drinking water directly from the river and tributaries. As a result of this direct exposure to the contaminated environment, there is a possibility that humans are consuming contaminated meat and milk products. Therefore, any type of contamination of the waterbodies is a major threat to the public health in Georgia through consumption of food produced using this water [8]. Contamination of the Mashavera River Basin, as one of the main regions contributing to the national food markets, raises multiple health concerns for the inhabitants of Georgia [25]. 

In addition to wastewater and dust particles from mining sites in the Mashavera River Basin, agricultural fertilizers are another source of heavy metal pollution [8]. Cd, Pb, Hg and As are toxic elements that have highly negative health consequences when consumed [25,69,72,81]. Some research studies have examined food contamination due to heavy metal toxicity [11,12,72]. In addition, commercial sediment extraction, wastewater discharge from local industries, as well as the outflow of untreated sewage in the Bolnisi and the Dmanisi regions are other causes for the contamination of water sources in the Mashavera River basin. Those activities should be properly monitored. Targeted collective action by all stakeholders should be taken to address these issues as part of a functioning water quality governance system. Full attention and responsiveness to maintaining environmental sustainability [82] must be given not only by the existing mining industries, but also by those overseeing the prospective mining activities in the Kvemo-Bolnisi village. 

## 4. Conclusions

Water quality of the Kazretula, Poladauri and Mashavera Rivers and three irrigation canals were examined in this research study during the HPS and LPS of 2016. The sediment and water analyses showed alarming levels of heavy metal contamination that exceed national and international thresholds in several observed sites of the Mashavera River Basin. High concentrations of Cd and Pb could be observed in the sediment samples as well in the water samples. The application of multiple indices to assess heavy metals in the study area indicate that the irrigation canals at sites 8, 12 and 17 have a contaminated status, with high levels of Pb, Cd, Cu, Zn and Ni. The Enrichment Factor results prove that the downstream areas of the Mashavera River, Poladauri River and the observed irrigation canals have anthropogenic enrichment of Cd, Cu, Zn and Pb. The average concentration of As, Cd and Pb were relatively high in the water samples at most sites. Based on the results of this research, more frequent spatial and temporal monitoring of multiple trace metal elements along the waterways, including the irrigation canals, is highly recommended to the respective authorities. By analyzing the heavy metals in the proximate water sources for the local crop and livestock production, indications of the negative impacts of poor water quality on the food production in the region were observed. However, this research is only a preliminary assessment of heavy metal contamination of the Mashavera River Basin. Therefore, an extensive study of possible effects of heavy metal contamination on dairy and meat products or the local small-scale fishing in this region is another research outlook. Furthermore, a comprehensive examination of the health status in the region as a result of heavy metal contamination is another timely and greatly needed area for research. Additionally, further research scrutinizing the causes behind diurnal and seasonal changes in heavy metal concentrations is recommended. 

## Figures and Tables

**Table 1:** Description of the sampling sites.

Sample Sites	Explanation
S1	Mashavera River, upstream at starting point of one irrigation canal
S2	Upstream irrigation canal
S3	Tributary coming from the Sakdrisi mine sites
S4	Kazretula River
S5	Mashavera River, before confluence of Kazretula River
S6	Javshaniani-Kveshi village tributary
S7	Mashavera River at Javshaniani-Kveshi
S8	Irrigation canal at Nakaduli village
S9	Tributary from Madneuli highland
S10	Poladauri River, upstream
S11	Irrigation canal in Poladauri village
S12	Irrigation canal in Vanati village
S13	Poladauri River, downstream
S14	After confluence of Poladauri River and Mashavera River at Rachisubani village
S15	Starting point of one irrigation canal off the Mashavera River
S16	Mashavera River, downstream at Khidiskuri village
S17	Irrigation canal at Tashtikulari village

**Table 2:** Sediment quality classification for multiple indices to assess heavy metals.

Geo-Accumulation Index (I_geo_) ^1^			Enrichment Factor (EF) ^2^		Contamination Factor (CF) ^3^		Pollution Load Index (PLI) ^4^	
I	I_geo_ Class	Sediment quality	EF Value	Nature of enrichment	CF Value	Pollution level	PLI	Indication
>0	0	Not polluted	EF < 2	Deficiency to mineral enrichment *	CF < 1	Low	0	Perfection
>0–1	1	Not polluted to moderately polluted	EF = 2–5	Moderate enrichment	1 ≤ CF ≥ 3	Moderate	<1	Baseline level
>1–2	2	Moderately polluted	EF = 5–20	Significant enrichment	3 ≤ CF ≥ 6	Considerable	>1	Polluted
>2–3	3	Moderately polluted to strongly polluted	EF = 20–40	Very high enrichment	CF > 6	Very high		
>3–4	4	Strongly polluted	EF > 40	Extremely high enrichment				
>4–5	5	Strongly polluted to extremely polluted						
>5	6	Extremely polluted						

* Zhang and Liu [51] identified 1.5 EF as the threshold value for natural and anthropogenic enrichment ^1^ Müller [19]; ^2^ Liu et al. [42]; ^3^ Goher et al. [41]; ^4^ Goher et al. [41].

**Table 3:** Classification of water quality based on the Metal Index (MI) ^1^.

MI	Class	Nature of Water Quality
<0.3	I	Very pure
0.3–1.0	II	Pure
1.0–2.0	III	Slightly affected
2.0–4.0	IV	Moderately affected
4.0–6.0	V	Strongly affected
>6.0	VI	Seriously affected

^1^ Ojekunle et al. [52].

**Table 4:** Variation of chemical properties (pH and EC).

Site	Water Analysis (In Situ) *				Sediment Analysis (Laboratory)			
	HPS		LPS		HPS		LPS	
	pH	EC (µS/cm)	pH	EC (µS/cm)	pH	EC (µS/cm)	pH	EC (µS/cm)
S1	8.46	109	8.45	194	7.62	500	7.78	267
S2	7.96	115	8.43	194	7.96	340	7.76	268
S3	7.76	320	8.48	594	8.06	280	8.32	272
S4	7.55	580	7.89	885	7.57	580	3.83	779
S5	8.12	127	8.33	275	7.54	590	7.71	269
S6	8.43	415	8.49	656	7.62	720	7.89	356
S7	8.13	164	8.34	6809	7.67	650	7.74	326
S8	7.81	142	8.41	279	7.64	470	7.59	455
S9	4.24	29,952	3.18	11,115	3.35	1440	2.98	2440
S10	8.91	1704	8.41	325	7.96	260	7.88	207
S11	8.73	166	8.44	297	7.95	160	7.86	315
S12	8.54	218	8.30	524	7.73	330	7.54	577
S13	8.70	237	8.01	542	7.52	560	7.74	330
S14	8.37	279	7.98	964	7.84	430	7.90	235
S15	8.24	301	8.28	586	7.85	570	7.63	610
S16	8.45	328	8.28	562	7.59	1070	7.63	775
S17	8.45	265	8.35	505	7.96	320	7.70	841

* Averages of morning, midday and evening water samples for each site.

**Table 5:** Heavy metal concentrations in sediments (mg/kg) at all sample sites in both seasons.

Sample Site	Pb		Cd		Cu		Cr		Ni		Hg		Zn		Mn		Fe	
	HPS	LPS	HPS	LPS	HPS	LPS	HPS	LPS	HPS	LPS	HPS	LPS	HPS	LPS	HPS	LPS	HPS	LPS
S1	18.1	14.3	0.292	0.132	41.3	32.2	35.8	30.3	29.2	21.9	0.03	0.01	102	84.4	923	738	40,500	44,700
S2	14.4	18.1	0.196	0.173	38.5	36.3	31.8	36.6	26.4	23.3	0.02	0.02	91.8	92.9	832	943	39,300	46,500
S3	186	130	1.55	1.88	1070	940	9.38	11.1	8.08	8.86	0.07	0.06	251	257	663	729	40,000	38,200
S4	94.9	73.9	2.4	1.97	1920	1640	7.44	4.93	14.4	7.29	0.05	0.06	752	614	667	499	55,100	58,700
S5	27.4	19.2	0.398	0.238	118	57	29.9	30.5	23.7	21.1	0.03	0.01	129	99.4	1100	888	41,500	44,700
S6	21	20.2	0.363	0.264	80.8	40.6	22.6	12.8	18.7	11.9	0.02	0.01	120	96.4	838	733	34,100	30,800
S7	23.2	25.9	0.89	1.21	212	311	21.1	22.4	16.9	18.4	0.02	0.02	196	260	814	820	36,900	39,600
S8	34.1	26.2	1.87	1.3	479	368	34.3	30.6	28.3	22.8	0.04	0.02	354	279	1130	1070	41,600	44,100
S9	13.4	12.7	1.63	3.35	460	430	22.4	47.4	16.3	34.1	0.02	0.02	537	1030	484	637	81,800	62,800
S10	11.1	11.8	0.105	0.155	43.2	34.6	29.2	25.8	22.5	19.8	0.01	0.01	80.8	78.7	664	701	46,700	44,300
S11	9.29	14.3	0.159	0.156	29.5	37.1	19	37.9	13.8	30.2	0.01	0.02	70.4	86.6	524	816	33,600	43,600
S12	25.2	23.5	8.35	6.11	666	419	39	35.4	33.8	30.5	0.04	0.03	2430	1860	1450	1200	41,900	39,100
S13	22.6	17	2.59	2.86	310	134	33.1	34.7	24.6	25.8	0.03	0.02	687	929	752	911	39,100	45,500
S14	20.8	16.7	1.7	0.936	319	292	24.8	16.7	24.1	16.9	0.02	0.01	461	355	888	687	40,300	34,000
S15	19.2	17.8	1.74	1.15	330	253	24	21.5	21.2	19.3	0.02	0.02	479	321	917	806	38,100	35,500
S16	28.8	25.9	2.44	1.43	406	261	38.7	36.4	31.1	29.8	0.04	0.03	476	258	977	1140	42,200	38,800
S17	63.4	35.3	2.01	2.78	458	541	27	41.8	26.5	36.5	0.09	0.04	584	494	853	1280	36,000	41,100
Mean	37.2	29.6	1.7	1.5	410.7	342.8	26.4	28.0	22.3	22.3	0.0	0.0	458.9	423.3	851.5	858.7	42,864.7	43,058.8
SD±	43.8	29.6	1.9	1.6	474.4	411.3	9.1	11.7	6.9	8.4	0.0	0.0	556.1	468.7	234.9	211.3	11,181.8	7996.7
STRV	46.7		1.2		34		81		20.9		0.15		150		N.S		N.S	

N.S: Not stated; SD: Standard Deviation; STRV: Sediment Toxicity Reference Values (STRVs) [58].

**Table 6:** Correlation matrix of heavy metals in the sediment samples for the HPS (Spearman correlation-*rs*).

Variable	Pb	Cd	Cu	Cr	Ni	Hg	Zn	Mn	Fe	PLI
Pb	1.000									
Cd	0.620 **	1.000								
Cu	0.733 **	0.765 **	1.000							
Cr	−0.012	0.277	−0.113	1.000						
Ni	0.108	0.380	−0.012	0.951 **	1.000					
Hg	0.903 **	0.569 *	0.652 **	0.179	0.298	1.000				
Zn	0.529 *	0.939 **	0.806 **	0.096	0.211	0.495 *	1.000			
Mn	0.324	0.375	0.113	0.735 **	0.792 **	0.370	0.199	1.000		
Fe	0.118	0.248	0.453	0.248	0.189	0.145	0.306	0.091	1.000	
PLI	0.789 **	0.926 **	0.895 **	0.174	0.321	0.754 **	0.885 **	0.363	0.328	1.000

** The correlation is significant at the 0.01 level (two-sided); * The correlation is significant at the 0.05 level (two-sided); PLI: Pollution Load Index

**Table 7:** Correlation matrix of heavy metals in the sediment samples for the LPS (Spearman correlation-*rs*).

Variable	Pb	Cd	Cu	Cr	Ni	Hg	Zn	Mn	Fe	PLI
Pb	1.000									
Cd	0.461	1.000								
Cu	0.679 **	0.826 **	1.000							
Cr	−0.275	0.186	−0.100	1.000						
Ni	−0.242	0.294	−0.037	0.968 **	1.000					
Hg	0.645 **	0.732 **	0.792 **	0.216	0.265	1.000				
Zn	0.280	0.909 **	0.750 **	0.132	0.245	0.537 *	1.000			
Mn	0.328	0.203	−0.029	0.561 *	0.613 **	0.222	0.108	1.000		
Fe	−0.244	0.052	−0.063	0.386	0.300	0.163	0.064	−0.101	1.000	
PLI	0.514 *	0.939 **	0.868 **	0.248	0.348	0.843 **	0.838 **	0.260	0.104	1.000

** The correlation is significant at the 0.01 level (two-sided); * The correlation is significant at the 0.05 level (two-sided); PLI: Pollution Load Index

**Table 8:** Average heavy metal concentrations in water samples (µg/L) for the HPS and LPS as well as various heavy metal concentration thresholds.

Sample Site	Cr		Ni		Cu		Zn		As		Cd		Pb	
	HPS	LPS	HPS	LPS	HPS	LPS	HPS	LPS	HPS	LPS	HPS	LPS	HPS	LPS
S1	0.7	50.5	0.9	46.4	1.0	47.3	3.1	109.6	0.4	28.2	1.3	0.9	15.8	11.1
S2	0.7	44.8	0.9	35.5	1.0	43.3	3.4	111.9	0.3	28.4	0.5	0.5	4.1	9.3
S3	0.3	28.1	0.5	34.0	40.5	2570.8	14.5	737.5	4.7	178.8	0.6	0.1	4.9	69.6
S4	0.5	24.2	1.5	41.7	260.4	7905.7	153.4	5380.1	4.2	307.6	1.6	0.0	6.0	62.8
S5	1.0	14.1	0.8	10.5	3.7	31.4	3.1	32.7	0.6	14.8	0.5	0.2	2.3	5.7
S6	0.8	5.9	0.5	2.7	2.8	10.0	3.5	24.2	0.8	9.9	0.5	0.4	1.0	1.4
S7	2.3	8.3	2.2	4.5	22.9	22.3	21.9	33.4	1.7	12.3	0.4	0.4	2.6	5.4
S8	1.7	14.0	1.4	11.8	21.5	107.6	18.2	121.8	1.3	16.1	0.3	0.1	2.3	5.5
S9	0.8	1.9	22.8	180.3	2603.7	13,157.6	21,912.2	125,057.1	0.6	5.4	62.5	0.8	0.2	0.9
S10	0.2	BD	2.6	BD	503.8	3.0	2342.7	BD	0.1	BD	10.1	BD	0.6	BD
S11	0.1	BD	0.0	BD	0.7	3.0	1.1	BD	0.1	BD	0.0	BD	0.2	BD
S12	0.1	BD	0.1	BD	8.2	0.8	19.5	BD	0.1	BD	0.1	BD	0.6	0.1
S13	0.1	BD	0.1	BD	8.4	3.3	20.3	7.7	0.1	BD	0.1	BD	0.4	0.0
S14	1.0	BD	0.7	BD	22.9	8.9	18.6	30.0	0.6	0.2	0.2	0.1	1.5	0.1
S15	0.5	BD	0.3	BD	21.4	4.1	8.5	BD	0.4	BD	0.1	BD	1.6	0.1
S16	1.1	BD	0.6	BD	15.5	3.5	9.1	BD	0.5	0.2	0.1	BD	0.9	0.1
S17	2.0	BD	1.3	BD	34.5	3.1	18.3	BD	1.0	BD	0.2	BD	2.6	0.2
Mean	0.8	21.3	2.2	40.8	210.2	1407.4	1445.4	11,967.8	1.0	54.7	4.6	0.4	2.8	11.5
SD±	0.7	17.1	5.4	54.9	630.2	3610.6	5304.1	37,541.0	1.4	98.0	15.1	0.3	3.7	22.5
MAC	500		100		1000		1000		50		1		30	
EU	50		20		2000		N.S		10		5		10	
WHO	5		70		2000		3000		10		3		10	
SWTRV	117.32		87.71		6.54		120		120		0.66		1.32	

BD—below the detection limit; SD: Standard deviation; N.S: Not stated; MAC: Maximum allowable concentration [65]; EU: The quality of water intended for human consumption by European Council Directive 98/83/EC [66]; WHO: World Health Organization Guidelines for drinking-water quality [67]; SWTRV: Surface Water Toxicity Reference Values (SWTRVs) [58].

**Table 9:** Heavy metals concentrations in the Mashavera River Basin according to other studies.

Reference	Sample Sites		Sample Type and Unit	Pb	Cd	Cu	Cr	Ni	Hg	Zn	Mn	As	Fe
	Other Studies	This Study											
Avkopashvili et al. [12]	N1	S5	Sediment (mg/kg)	N.S	0.004	0.01	N.S	N.S	N.S	0.04	N.S	N.S	0.12
	N2	S4		N.S	13.2	1.4	N.S	N.S	N.S	510.2	N.S	N.S	1.1
	N5	S14		N.S	0.3	1.6	N.S	N.S	N.S	26.6	N.S	N.S	18.2
	N6	S16		N.S	0.05	0.3	N.S	N.S	N.S	1.7	N.S	N.S	0.6
Hanauer et al. [79]	Top soil (0–20 cm) in Mashavera valley, irrigated contaminated	C.T	Topsoil (mg/kg)	N.S	1.73	314.99	N.S	N.S	N.S	343.68	N.S	N.S	N.S
Melikadze [74]	N1	S4	Water (mg/L)	0.34	21.6	2334.7	N.S	N.S	N.S	N.S	71.7	N.S	1552.0
	N3	S9		0.5	10	1680	N.S	0.14	N.S	372	101	N.S	1152.0
	N6	S5		0.2	0.14	32	N.S	N.S	N.S	9.84	1.96	N.S	9.6
	N7	S7		0.05	0.04	16	N.S	N.S	N.S	7.08	0.98	N.S	6.4
	N10	S14		N.S	N.S	8.1	N.S	N.S	N.S	5.10	1.0	N.S	2.4
NEA 2013 [80]	Lower Mashavera	C.T	Water (mg/L)	N.S	N.S	1.2	N.S	N.S	N.S	N.S	0.12	N.S	1.31
NEA 2014 [80]	Lower Mashavera	C.T	Water (mg/L)	0.0536	N.S	N.S	N.S	N.S	N.S	N.S	1.04	N.S	1.73
NEA 2015 [80]	Upper Mashavera	C.T	Water (mg/L)	0.0412	0.0083	N.S	N.S	0.1057	N.S	N.S	0.2817	N.S	0.6783

N.S: Not stated; C.T: Cannot be precisely located; NEA: National Environmental Agency of Georgia.

## Supplementary Materials

The following are available online at www.mdpi.com/1660-4601/15/4/621, Figure S1: Geo-accumulation Index (Igeo) values of heavy metals in sediments of samples sites, Figure S2: Enrichment Factors (EF) of heavy metals in sediments of samples sites, Figure S3: Contamination Factors (CF) of heavy metals in sediments of the samples sites, Figure S4: Spatial assessment of Pollution Load Index (PLI) for both seasons (data for the geographical analysis) Figure S5: Diurnal trends in heavy metal concentration for selected sample sites, Figure S6: MI values for morning, midday and evening water samples for both seasons.
