# Supplementary material for: Water Quality in Surface Water: A Preliminary Assessment of Heavy Metal Contamination of the Mashavera River, Georgia
Source: Int J Environ Res Public Health. 2018 Mar 28;15(4):621. doi: 10.3390/ijerph15040621 (PMC5923663; doi:10.3390/ijerph15040621)
Supplement: Supplementary file 1 [file ijerph-15-00621-s001.pdf]

Supplementary Materials

Water Quality in Surface Water: A Preliminary Assessment of Heavy Metal Contamination of the Mashavera River, Georgia

Sisira S. Withanachchi [1,*], Giorgi Ghambashidze [2], Ilia Kunchulia [3], Teo Urushadze [2], Angelika Ploeger [1]

[1] Department of Organic Food Quality and Food Culture, Faculty of Organic Agricultural Sciences, University of Kassel, 37213 Witzenhausen, Germany; a.ploeger@uni-kassel.de
[2] School of Agricultural and Natural Sciences, Agricultural University of Georgia, Tbilisi 0159, Georgia; g.ghambashidze@agruni.edu.ge (G.G.); t.urushadze@agruni.edu.ge (T.U.)
[3] Michail Sabashvili Institute of Soil Science, Agrochemistry and Melioration, Agricultural University of Georgia, Tbilisi 0159, Georgia; iliakunchulia@gmail.com
* Correspondence: sisirawitha@uni-kassel.de; Tel.: +49-055-4298-1627

Abbreviation
HPS: High precipitation season
LPS: Low precipitation season

Figure S1: Geo-accumulation Index (I_{geo}) values of heavy metals in sediments of samples sites.

a). Geo-accumulation (I_{geo}) in HPS

Sites	Pb	Cd	Cu	Ni	Hg	Zn	Mn	Fe
**S1**	-0.73	-0.62	-0.71	-1.80	-4.22	-0.48	-0.47	-0.66
**S2**	-1.06	-1.20	-0.81	-1.95	-4.95	-0.63	-0.62	-0.70
**S3**	2.63	1.78	3.99	-3.66	-3.10	0.82	-0.94	-0.68
**S4**	1.66	2.42	4.83	-2.82	-3.46	2.40	-0.93	-0.22
**S5**	-0.13	-0.18	0.81	-2.11	-4.26	-0.14	-0.21	-0.63
**S6**	-0.51	-0.31	0.26	-2.45	-4.80	-0.25	-0.61	-0.91
**S7**	-0.37	0.98	1.65	-2.59	-4.80	0.46	-0.65	-0.80
**S8**	0.18	2.06	2.83	-1.85	-4.03	1.31	-0.17	-0.62
**S9**	-1.16	1.86	2.77	-2.65	-5.16	1.91	-1.40	0.35
**S10**	-1.43	-2.10	-0.64	-2.18	-5.37	-0.82	-0.94	-0.46
**S11**	-1.69	-1.50	-1.19	-2.89	-6.04	-1.02	-1.28	-0.93
**S12**	-0.25	4.21	3.30	-1.59	-4.04	4.09	0.19	-0.61
**S13**	-0.41	2.52	2.20	-2.05	-4.37	2.27	-0.76	-0.71
**S14**	-0.53	1.92	2.24	-2.08	-5.17	1.69	-0.52	-0.67
**S15**	-0.64	1.95	2.29	-2.27	-5.05	1.75	-0.48	-0.75
**S16**	-0.06	2.44	2.59	-1.71	-4.10	1.74	-0.38	-0.60
**S17**	1.08	2.16	2.76	-1.94	-2.75	2.04	-0.58	-0.83

b) Geo-accumulation (I_{geo}) in LPS

Sites	Pb	Cd	Cu	Ni	Hg	Zn	Mn	Fe
**S1**	-1.07	-1.77	-1.07	-2.22	-5.35	-0.76	-0.79	-0.52
**S2**	-0.73	-1.38	-0.89	-2.13	-5.25	-0.62	-0.44	-0.46
**S3**	2.12	2.06	3.80	-3.53	-3.36	0.85	-0.81	-0.75
**S4**	1.30	2.13	4.60	-3.81	-3.35	2.11	-1.35	-0.13
**S5**	-0.64	-0.92	-0.24	-2.27	-5.35	-0.52	-0.52	-0.52
**S6**	-0.57	-0.77	-0.73	-3.10	-5.71	-0.56	-0.80	-1.06
**S7**	-0.21	1.43	2.20	-2.47	-5.09	0.87	-0.64	-0.69
**S8**	-0.20	1.53	2.45	-2.16	-4.77	0.97	-0.25	-0.54
**S9**	-1.24	2.90	2.67	-1.58	-4.64	2.85	-1.00	-0.03
**S10**	-1.35	-1.54	-0.96	-2.36	-5.71	-0.86	-0.86	-0.53
**S11**	-1.07	-1.53	-0.86	-1.76	-4.84	-0.72	-0.64	-0.55
**S12**	-0.35	3.76	2.63	-1.74	-4.51	3.71	-0.09	-0.71
**S13**	-0.82	2.67	0.99	-1.98	-4.91	2.70	-0.48	-0.49
**S14**	-0.85	1.06	2.11	-2.59	-5.57	1.32	-0.89	-0.91
**S15**	-0.75	1.35	1.91	-2.40	-5.25	1.17	-0.66	-0.85
**S16**	-0.21	1.67	1.95	-1.78	-4.41	0.86	-0.16	-0.72
**S17**	0.23	2.63	3.00	-1.48	-3.80	1.79	0.01	-0.64

Figure S2: Enrichment Factors (EF) of heavy metals in sediments of samples sites.

**HPS - Pb**	
**Site**	**EF**
**S1**	1.05471605
**S2**	0.86473282
**S3**	10.974
**S4**	4.0646824
**S5**	1.55816867
**S6**	1.45337243
**S7**	1.48379404
**S8**	1.93451923
**S9**	0.38660147
**S10**	0.56094218
**S11**	0.6525119
**S12**	1.41937947
**S13**	1.36409207
**S14**	1.21806452
**S15**	1.18929134
**S16**	1.61061611
**S17**	4.15622222

LPS-Pb	
**Site**	**EF**
**S1**	0.75498881
**S2**	0.91862366
**S3**	8.03141361
**S4**	2.97110733
**S5**	1.01369128
**S6**	1.54779221
**S7**	1.54353535
**S8**	1.40208617
**S9**	0.47726115
**S10**	0.62862302
**S11**	0.7740367
**S12**	1.41841432
**S13**	0.88175824
**S14**	1.15917647
**S15**	1.18332394
**S16**	1.57536082
**S17**	2.02695864

HPS-Cd	
**Site**	**EF**
**S1**	1.13435391
**S2**	0.78466497
**S3**	6.09666667
**S4**	6.85299456
**S5**	1.50888353
**S6**	1.67483871
**S7**	3.79476061
**S8**	7.0724359
**S9**	3.13512632
**S10**	0.35374732
**S11**	0.74452381
**S12**	31.3540175
**S13**	10.4218244
**S14**	6.63688999
**S15**	7.18530184
**S16**	9.09699842
**S17**	8.78444444

LPS-Cd	
**Site**	**EF**
**S1**	0.4646085
**S2**	0.58534767
**S3**	7.74310646
**S4**	5.28018171
**S5**	0.83770321
**S6**	1.34857143
**S7**	4.80740741
**S8**	4.63794407
**S9**	8.39278132
**S10**	0.55048909
**S11**	0.56293578
**S12**	24.5858483
**S13**	9.88952381
**S14**	4.33129412
**S15**	5.09671362
**S16**	5.79862543
**S17**	10.6420114

HPS-Cu	
**Site**	**EF**
**S1**	1.06960768
**S2**	1.02753746
**S3**	28.0577778
**S4**	36.5493043
**S5**	2.98238286
**S6**	2.48534376
**S7**	6.02613671
**S8**	12.0773504
**S9**	5.89839717
**S10**	0.97027837
**S11**	0.92089947
**S12**	16.6720764
**S13**	8.31599886
**S14**	8.30261924
**S15**	9.08486439
**S16**	10.0912059
**S17**	13.3441975

LPS-Cu	
**Site**	**EF**
**S1**	0.75557544
**S2**	0.81881004
**S3**	25.8103549
**S4**	29.3045618
**S5**	1.33750932
**S6**	1.38262626
**S7**	8.23748597
**S8**	8.75263291
**S9**	7.18188252
**S10**	0.81922247
**S11**	0.89251784
**S12**	11.2400114
**S13**	3.08903541
**S14**	9.00810458
**S15**	7.47517997
**S16**	7.0556701
**S17**	13.8065423

HPS-Cr	
**Site**	**EF**
**S1**	0.46358299
**S2**	0.42435963
**S3**	0.12298222
**S4**	0.07081428
**S5**	0.37785274
**S6**	0.34757902
**S7**	0.29988558
**S8**	0.43241453
**S9**	0.14361315
**S10**	0.32791815
**S11**	0.29656085
**S12**	0.48814638
**S13**	0.44396704
**S14**	0.32273504
**S15**	0.33035871
**S16**	0.48094787
**S17**	0.39333333

LPS-Cr	
**Site**	**EF**
**S1**	0.3554959
**S2**	0.41278853
**S3**	0.15239092
**S4**	0.04404619
**S5**	0.35784241
**S6**	0.21795094
**S7**	0.29665544
**S8**	0.36390023
**S9**	0.39583864
**S10**	0.30543266
**S11**	0.45588175
**S12**	0.47481671
**S13**	0.39996093
**S14**	0.25759477
**S15**	0.31762128
**S16**	0.49200458
**S17**	0.53337659

HPS-Ni	
**Site**	**EF**
**S1**	0.50045025
**S2**	0.4662775
**S3**	0.14021176
**S4**	0.1814028
**S5**	0.39639972
**S6**	0.38064516
**S7**	0.31790212
**S8**	0.47220023
**S9**	0.1383144
**S10**	0.33442499
**S11**	0.28508403
**S12**	0.55993261
**S13**	0.43670829
**S14**	0.41509269
**S15**	0.38622819
**S16**	0.51154168
**S17**	0.51094771

**LPS-Ni**	
**Site**	**EF**
**S1**	0.34007106
**S2**	0.34780519
**S3**	0.16099168
**S4**	0.08620303
**S5**	0.32764837
**S6**	0.26818182
**S7**	0.32251931
**S8**	0.35886355
**S9**	0.37690146
**S10**	0.31023768
**S11**	0.48078791
**S12**	0.54144727
**S13**	0.39358759
**S14**	0.3450173
**S15**	0.37736537
**S16**	0.53311098
**S17**	0.61643051

**HPS-Hg**	
**Site**	**EF**
**S1**	0.09410864
**S2**	0.05824936
**S3**	0.2065
**S4**	0.11714338
**S5**	0.08928193
**S6**	0.07474487
**S7**	0.06907317
**S8**	0.10410096
**S9**	0.02423472
**S10**	0.03663812
**S11**	0.03195833
**S12**	0.10307399
**S13**	0.08782097
**S14**	0.04889826
**S15**	0.05605774
**S16**	0.0978673
**S17**	0.29205

LPS-Hg	
**Site**	**EF**
**S1**	0.03880537
**S2**	0.04009462
**S3**	0.18008901
**S4**	0.11820102
**S5**	0.03880537
**S6**	0.04405844
**S7**	0.05244444
**S8**	0.05886621
**S9**	0.04528344
**S10**	0.03063205
**S11**	0.05683486
**S12**	0.07967263
**S13**	0.05160879
**S14**	0.04372941
**S15**	0.05251831
**S16**	0.08606701
**S17**	0.12345499

HPS-Zn	
**Site**	**EF**
**S1**	1.25130604
**S2**	1.16056247
**S3**	3.11768421
**S4**	6.78085777
**S5**	1.54440076
**S6**	1.74841797
**S7**	2.63905292
**S8**	4.22793522
**S9**	3.26166517
**S10**	0.85963259
**S11**	1.04100251
**S12**	28.8144705
**S13**	8.72968098
**S14**	5.68347917
**S15**	6.24638762
**S16**	5.60419057
**S17**	8.05988304

LPS-Zn	
**Site**	**EF**
**S1**	0.93810903
**S2**	0.99261573
**S3**	3.34262882
**S4**	5.19695149
**S5**	1.10483457
**S6**	1.55505126
**S7**	3.26209463
**S8**	3.14328679
**S9**	8.14884345
**S10**	0.88265178
**S11**	0.98684693
**S12**	23.6349441
**S13**	10.1443146
**S14**	5.1876161
**S15**	4.49257228
**S16**	3.3037439
**S17**	5.97177616

HPS -Mn	
**Site**	**EF**
**S1**	1.26552215
**S2**	1.17558449
**S3**	0.9204
**S4**	0.67219814
**S5**	1.47186393
**S6**	1.36462308
**S7**	1.22495776
**S8**	1.50837104
**S9**	0.32856033
**S10**	0.78954024
**S11**	0.8659944
**S12**	1.92166222
**S13**	1.06798255
**S14**	1.22357612
**S15**	1.33649529
**S16**	1.28559799
**S17**	1.31573856

LPS-Mn	
**Site**	**EF**
**S1**	0.91679432
**S2**	1.12611259
**S3**	1.0597105
**S4**	0.4720473
**S5**	1.10313462
**S6**	1.32152788
**S7**	1.14985146
**S8**	1.34731226
**S9**	0.56325215
**S10**	0.8786934
**S11**	1.03926606
**S12**	1.70422747
**S13**	1.11180866
**S14**	1.12202076
**S15**	1.26075228
**S16**	1.63153426
**S17**	1.72938314

Figure S3: Contamination Factors (CF) of heavy metals in sediments of the samples sites

	Pb	Cd	Cu	Cr	Ni	Hg	Zn	Mn	Fe
**HPS**	1.9	5.6	9.1	0.3	0.3	0.1	4.8	1.0	1.0
**LPS**	1.5	5.1	7.6	0.3	0.3	0.1	4.5	1.0	1.0

Figure S4: Spatial assessment of Pollution Load Index (PLI) for both seasons (Data for the geographical analysis)

	S1	S2	S3	S4	S5	S6	S7	S8	S9	S10	S11	S12	S13	S14	S15	S16	S17
**HPS**	0.8	0.7	1.1	1.2	0.9	0.8	0.9	1.1	1.0	0.6	0.6	1.4	1.1	1.0	1.0	1.1	1.2
**LPS**	0.7	0.7	1.1	1.1	0.7	0.6	0.9	1.0	1.2	0.6	0.7	1.3	1.0	0.8	0.9	1.0	1.2

Figure S5: Diurnal trends in heavy metal concentration for selected sample sites

Site	HPS	Cr	Ni	Cu	Zn	As	Cd	Pb
4	Morning	0.117	0.353	17.249	4.847	1.452	0.787	3.856
	Mid-day	0.290	1.003	145.429	138.903	1.023	1.161	4.082
	Evening	0.963	2.997	618.613	316.456	10.024	2.789	10.120

	**LPS**	Cr	Ni	Cu	Zn	As	Cd	Pb
**4**	Morning	39.391	70.957	15894.997	8838.074	600.656	0.028	125.037
	Mid-day	4.902	19.271	1867.622	3110.539	17.423	0.045	6.283
	Evening	28.409	34.963	5954.591	4191.604	304.812	0.038	57.062
	**HPS**	Cr	Ni	Cu	Zn	As	Cd	Pb
**3**	Morning	0.597	0.979	91.287	33.639	11.451	0.424	8.628
	Mid-day	0.122	0.298	14.179	5.084	1.225	0.555	2.248
	Evening	0.104	0.349	16.111	4.770	1.416	0.765	3.704
	**LPS**	Cr	Ni	Cu	Zn	As	Cd	Pb
**3**	Morning	26.165	30.281	2252.744	567.977	157.023	0.019	68.372
	Mid-day	56.263	66.799	4972.116	1575.770	355.672	0.072	136.604
	Evening	1.795	4.795	487.455	68.806	23.852	0.079	3.848
	**HPS**	Cr	Ni	Cu	Zn	As	Cd	Pb
**8**	Morning	0.646	0.470	4.057	3.623	0.756	0.193	3.471
	Mid-day	2.503	2.242	30.324	27.464	1.714	0.442	2.032
	Evening	1.889	1.606	30.146	23.480	1.442	0.247	1.303
	**LPS**	Cr	Ni	Cu	Zn	As	Cd	Pb
**8**	Morning	17.277	13.967	92.314	130.846	17.649	0.032	4.683
	Mid-day	11.994	10.754	136.393	120.006	13.482	0.180	6.891
	Evening	12.745	10.653	93.997	114.514	17.123	0.080	4.858
	**HPS**	Cr	Ni	Cu	Zn	As	Cd	Pb
**9**	Morning	0.641	24.753	2334.527	24439.383	0.640	57.082	0.163
	Mid-day	0.691	27.917	3097.300	26979.263	0.651	79.207	0.216
	Evening	1.125	15.675	2379.326	14317.962	0.466	51.178	0.238
	**LPS**	Cr	Ni	Cu	Zn	As	Cd	Pb
**9**	Morning	3.420	331.501	33256.770	335121.122	9.079	0.855	0.937
	Mid-day	0.461	29.066	5836.962	38701.317	1.795	0.244	0.847
	Evening	BD	0.000	379.207	1348.929	BD	1.181	BD
	**HPS**	Cr	Ni	Cu	Zn	As	Cd	Pb
**14**	Morning	1.326	0.798	34.561	21.310	0.762	0.221	1.679
	Mid-day	1.247	1.078	29.141	30.214	0.733	0.202	2.204
	Evening	0.278	0.145	5.143	4.174	0.158	0.054	0.575
	**HPS**	Cr	Ni	Cu	Zn	As	Cd	Pb
**15**	Morning	1.061	0.734	53.004	19.585	0.919	0.224	3.493

	Mid-day	0.162	0.081	4.056	1.742	0.129	0.038	0.758
	Evening	0.344	0.216	7.274	4.084	0.207	0.055	0.538
	**HPS**	Cr	Ni	Cu	Zn	As	Cd	Pb
**16**	Morning	0.348	0.220	7.356	4.115	0.218	0.056	0.557
	Mid-day	2.539	1.470	34.165	18.766	1.126	0.175	1.365
	Evening	0.313	0.184	5.058	4.542	0.130	0.050	0.831
	**HPS**	Cr	Ni	Cu	Zn	As	Cd	Pb
**17**	Morning	1.548	1.009	41.539	17.793	0.975	0.238	3.402
	Mid-day	2.462	1.539	36.656	21.491	1.099	0.222	3.116
	Evening	2.110	1.264	25.358	15.638	0.813	0.169	1.277

Figure S6: MI values for morning, midday and evening water samples for both seasons

MI Morning

**Sample**	**MI HPS**	**MI LPS**
**S1**	1.01946306	4.51938578
**S2**	0.69009494	5.76315488
**S3**	2.22124737	24.3464906
**S4**	0.80880173	84.5644719
**S5**	0.39010371	2.51449194
**S6**	0.45915012	1.35614643
**S7**	1.12321479	1.9432463
**S8**	0.49818256	2.56788064
**S9**	28.7764355	134.36456
**S10**	13.2827951	0.00148145
**S11**	0.0258598	0
**S12**	0.13013568	0.00040289
**S13**	0.12380118	0.00085299
**S14**	0.35616104	0.06130088
**S15**	0.5616525	0.01278798
**S16**	0.10523378	0.00889956
**S17**	0.56106594	0.00115697

MI Mid-day

**Sample**	**MI HPS**	**MI LPS**
**S1**	1.64822769	5.29056605
**S2**	0.67931006	4.73723094
**S3**	0.54547813	53.3296533

**S4**	1.03144452	4.64126739
**S5**	0.81816514	2.09020265
**S6**	0.29636308	1.34082231
**S7**	0.21895468	1.83899855
**S8**	0.58339399	2.38304775
**S9**	37.4307263	16.5806992
**S10**	0.2041104	0
**S11**	0.05229082	0
**S12**	0.16671248	0.00639164
**S13**	0.07530973	0.00076945
**S14**	0.40358944	0.00169505
**S15**	0.10538817	0.00658803
**S16**	0.35690006	0.00890301
**S17**	0.54777418	0.01714014

MI Evening

**Sample**	**MI HPS**	**MI LPS**
**S1**	3.47040858	5.35426633
**S2**	0.51837433	3.34279003
**S3**	0.78187978	3.13502105
**S4**	3.40373999	41.1306913
**S5**	0.16435992	2.36773497
**S6**	0.32056173	1.18704567
**S7**	0.48896864	2.26086045
**S8**	0.40635542	2.48743262
**S9**	23.3181259	1.03286903
**S10**	0.04565111	0
**S11**	0.02958847	0
**S12**	0.07800048	0.00662983
**S13**	0.07945935	0.00961826
**S14**	0.09774172	0.04512971
**S15**	0.10161181	0.00756052
**S16**	0.12004396	0.01141178
**S17**	0.30556659	0.02280242
